# Metabolomics in neonatal sepsis: A critical appraisal of current evidence

**DOI:** 10.1016/j.csbj.2025.10.034

**Published:** 2025-10-18

**Authors:** Aliya Baizhanova, Zhalaliddin Makhammajanov, Dinara Galiyeva, Olga Mironova, Abduzhappar Gaipov, Vitaliy Sazonov

**Affiliations:** a Department of Medicine, School of Medicine, Nazarbayev University, Astana Z05K4F4, Kazakhstan; b Department of Biomedical Sciences, School of Medicine, Nazarbayev University, Astana Z05K4F4, Kazakhstan; c Pediatric Anesthesiology and Intensive Care Unit, National Research Center for Maternal and Child Health, “University Medical Center”, Astana Z05K4F4, Kazakhstan; d Department of Surgery, School of Medicine, Nazarbayev University, Astana Z05K4F4, Kazakhstan

**Keywords:** Sepsis, Neonatal sepsis, Metabolomics, Omics technology

## Abstract

Neonatal sepsis remains a leading cause of morbidity and mortality worldwide; however, timely and accurate diagnosis continues to present a significant clinical challenge. Current diagnostic methods, including blood culture and inflammatory markers, are limited by delayed turnaround times and a lack of specificity, often leading to overtreatment or missed opportunities for intervention. In recent years, metabolomics has emerged as a promising approach for discovering novel biomarkers that reflect the systemic metabolic disruptions characteristic of neonatal sepsis. This systematic review summarizes the existing evidence on changes in metabolites and metabolite classes associated with neonatal sepsis, with a focus on their potential as biomarkers. Eleven eligible studies were reviewed, covering various analytical techniques and biofluids, including serum, urine, and stool. Although a wide range of metabolite alterations was observed, particular attention was given to amino acid metabolism, energy substrates, and lipid derivatives. The findings emphasize both the potential and current limitations of metabolomics-based biomarker discovery in this area. The review identifies important gaps in the literature, including diverse study designs, small sample sizes, and inconsistent reporting, highlighting the need for rigorous, standardized research. The importance and clinical potential of metabolomics in neonatal sepsis are thoroughly discussed.

## Graphical Abstract

**Figure:**
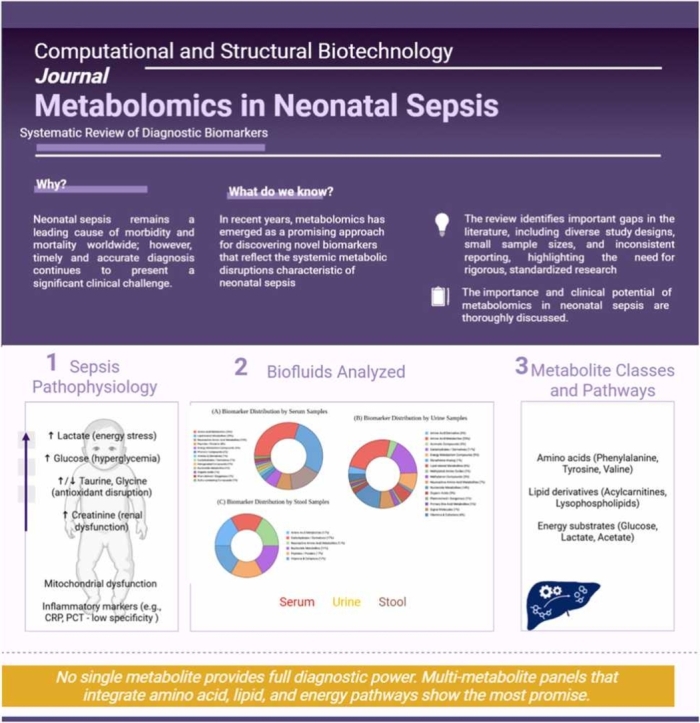

## 1 Introduction

Neonatal sepsis remains one of the most critical challenges in modern neonatology, contributing significantly to morbidity and mortality in neonatal intensive care units worldwide [1]. Despite advances in supportive care, the diagnosis of sepsis in neonates remains difficult due to often subtle and nonspecific early signs [2]. The therapeutic window for effective treatment is narrow: delays in diagnosis may result in multi-organ failure and poor outcomes, while unnecessary antibiotic use increases the risk of antimicrobial resistance and adverse effects [3]. Therefore, there is a strong need for fast, accurate diagnostic methods that can distinguish true sepsis from other neonatal conditions with similar symptoms.

Traditional diagnostic approaches, including blood cultures, C-reactive protein (CRP), and procalcitonin (PCT), face significant limitations [2], [4], [5]. Blood cultures, considered the gold standard, are hampered by delayed results and high false-negative rates, mainly because neonates often have low blood volumes sampled [2], [4], [5]. Biomarkers such as CRP and PCT lack the specificity necessary to reliably differentiate sepsis from other inflammatory states, although PCT has shown better performance [6], [7]. This diagnostic uncertainty often leads to the use of broad-spectrum antibiotics without a confirmed infection [3]. As a result, neonatologists frequently start empirical therapy, which can cause unnecessary drug exposure and resistance development.

Over the past decade, metabolomics has emerged as a groundbreaking field, providing an unbiased and detailed snapshot of metabolic changes associated with disease processes. Unlike genomics or proteomics, metabolomics provides a direct functional indication of how the host responds to infection, revealing alterations across various metabolic pathways [8]. In neonatal sepsis, this approach shows promise not only for identifying reliable biomarkers but also for understanding the disease’s underlying mechanisms [9], [10]. Previous studies have observed disturbances in amino acid, carbohydrate, and lipid metabolism during neonatal sepsis, with specific metabolites showing potential as early diagnostic or prognostic markers [9]. However, despite this potential, the field faces challenges such as methodological differences, small sample sizes, and inconsistent validation, which hinder widespread clinical use [11]. Importantly, variability in biofluid choices, analytical techniques, and definitions of sepsis across studies has led to conflicting findings, making it challenging to identify dependable biomarkers [11], [12], [13], [14]. Additionally, there is limited agreement on which metabolite classes best reflect sepsis pathophysiology, and the practical application of proposed biomarkers requires validation in larger, multicenter studies.

This systematic review aims to critically evaluate and synthesize existing evidence on metabolomic changes in neonatal sepsis, focusing on identifying and validating potential biomarkers across different biofluids and methods. By examining recurrent metabolites and their clinical significance, this review aims to clarify the current state of research and explore the opportunities and challenges of implementing metabolomics in the routine diagnosis of neonatal sepsis. This comprehensive view is essential for guiding future studies and ultimately improving outcomes for this vulnerable population.

## 2 Methods

**Fig. 1:**
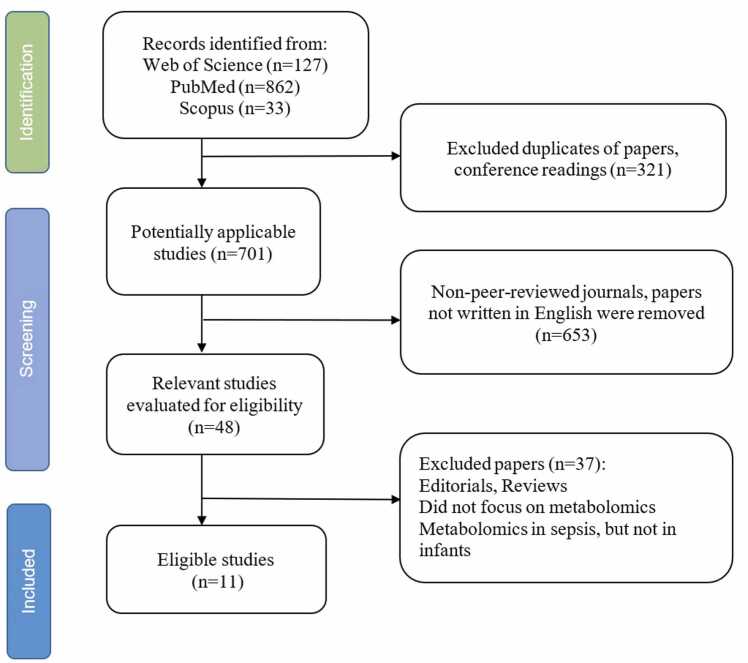
Flowchart of search strategy.

The objective of this systematic review was to identify metabolites or metabolite classes that are frequently altered in neonatal sepsis, providing new insights into their potential utility as biomarkers. A comprehensive literature search was conducted using the PubMed, Scopus, and Web of Science databases from their inception until June 25, 2025. The data were searched using terms such as “Neonatal sepsis,” “Metabolomics in neonatal sepsis,” and “Biomarkers of neonatal sepsis”. Additional studies were identified through manual searches of reference lists from relevant papers. Boolean operators ("AND" and "OR") were used to optimize the search (see the Supplemental materials). The initial search yielded 48 potentially relevant articles. The selection process is illustrated in Fig. 1, which follows the Preferred Reporting Items for Systematic Reviews and Meta-Analyses (PRISMA) guidelines [15].

Studies eligible for inclusion met the following criteria: original research articles; a neonatal population (age ≤28 days); a confirmed or suspected diagnosis of neonatal sepsis; and a metabolomic analysis performed on human biological samples. Studies were excluded if they were non-original (e.g., reviews, case reports, or letters), animal or in vitro studies, or lacked clearly defined metabolomic methods.

The extracted information included the following: study characteristics, metabolite name, biofluid of analysis, directionality of change (up or downregulation), fold change (if reported), and data on whether the metabolite was explicitly identified as a potential biomarker by the authors. Metabolites with unclear biomarker potential were explicitly categorized as "no." For metabolites that were not directly identified as biomarkers by the authors but demonstrated robust associations with sepsis, classification as potential biomarkers was applied cautiously.

All identified metabolites were mapped to metabolic classes using the Human Metabolome Database (HMDB) [16] and grouped into a broader class based on their metabolic pathway, thereby fostering a comprehensive understanding of how specific metabolite families are involved in sepsis-related metabolic changes.

## 3 Risk of bias and study quality assessment

We used QUADAS-2, adapted for discovery validation studies with a diagnostic accuracy focus, to assess study-level quality. We used ROBINS-I for observational, non-randomized designs. The domains included patient selection; the index test, including metabolomics workflow, pre-analytic handling, and blinding; the reference standard, including the sepsis definition; flow and timing; confounding control, including nutrition, antibiotics, and comorbidities; and reporting transparency, including feature selection and overfitting safeguards. Two reviewers performed independent assessments and resolved discrepancies through consensus.

## 4 Statistical analysis

The analysis was conducted among unique metabolites only, unless a different trend was observed in different studies. Frequency analysis was employed to identify the most frequently reported biomarkers among metabolites or metabolite classes. Given the small sample size, a metabolite was considered “frequent” if it was reported in two or more independent studies.

The overrepresentation analysis (ORA) was performed to investigate whether a specific metabolic family was disproportionately represented among all reported metabolites in neonatal sepsis. The nature of ORA allowed us to assess the proportion of potential biomarkers in each class that exceeded the expected chance relative to all other classes. Fisher’s exact test was used to test this statistically, providing exact *p*-values for the overrepresentation. *P*-values < 0.05 were deemed to be statistically significant. To extract the magnitude of overrepresentation, the enrichment ratios (ER) were calculated.

To ensure unbiased analysis, the threshold for overrepresentation was pre-specified. A class was significantly overrepresented if the enrichment ratio exceeded 1.3 (proportion of metabolites within a specific class at least 30 % higher than that in other classes) and had a Fisher’s exact test *p*-value < 0.05. In cases where the enrichment ratio was moderately high (above 1.1) but the *p*-value was not statistically significant, the decision was made to interpret the results as suggestive of enrichment. Given that metabolomic samples are small and underpowered, taking such caution allows for avoiding faulty overinterpretation and still highlights the interest in investigating them in the future.

## 5 Results

A total of 11 studies were included in the final analysis: five case-control studies, two cohort studies, and four prospective studies. These studies were conducted in various geographical regions, including Europe, North America, and Asia. They demonstrated variability in analytical techniques, such as Proton nuclear magnetic resonance (1H-NMR), Gas Chromatography-Mass Spectrometry (GC-MS), Ultra-Performance Liquid Chromatography-Tandem Mass Spectrometry (UPLC-MS/MS), liquid chromatography-tandem mass spectrometry (LC-MS/MS), Liquid Chromatography-Mass Spectrometry (LC-MS), Ultra-Performance Liquid Chromatography coupled with Quadrupole Time-of-Flight Mass Spectrometry (UPLC-QToF-MS), and Ultra-Performance Liquid Chromatography-Triple Quadrupole Mass Spectrometry (UPLC-TQ-MS). Most studies used serum and urine samples, while one study analyzed stool samples. Sample sizes varied significantly, ranging from small cohorts of four to large, population-based studies of 4794 participants. Table 1 contains the general characteristics of all included studies.

**Table 1:** Characteristics of the included studies.

**Study ID**	**First Author, Year, Country**	**Study Type**	**Biofluid blood-derived/urine/stool**	**Analytical Method**	**Sample Size (Sepsis)**	**Sample Size (Control)**	**Reported diagnostic performance (where available)**	**Sepsis Definition**
S1	Mickiewicz et al. [57], 2013, Canada/USA	Case-control (prospective)	Serum	1H-NMR spectroscopy (Targeted profiling)	60 (10 non-survivors)	40 SIRS + 40 Healthy	AUC= Diagnosis ∼0.98; mortality 0.91–1.00	Septic shock diagnosed clinically in PICU (with PRISM III-APS, PCT)
S2	Fanos et al. [28], 2014, Italy	Case-control (prospective, multi-center)	Urine	1H-NMR + GC-MS	9 (5 EOS, 4 LOS)	16		Clinical diagnosis (EOS ≤ 3–6 days; LOS > 4–7 days)
S3	Bekhof et al. [58], 2015, Netherlands	Prospective cohort	Urine	Semi-quantitative reagent strip (Combur3Test® Roche)	58 LOS episodes in 123 preterm infants	65 episodes classified as non-sepsis	Sens 69 %, Spec 54 %; LR 1.49- not clinically helpful alone.	LOS defined as clinical and/or blood culture-proven sepsis after 72 h
S4	Stewart et al. [59], 2016, UK	Case-control (longitudinal, matched)	Serum	Untargeted UPLC-MS/MS + Shotgun proteomics	10 LOS (longitudinal: pre, diag, post)	9 matched controls		Late-onset sepsis diagnosed by clinician, confirmed independently and blind reviewed
S5	Fell et al. [60], 2017, Canada	Population-based cohort	Dried blood spot (heel prick)	Mass spectrometry + enzymatic assays	4794 term/late preterm	788,344		ICD−10-CA code P36 in hospitalization records
S6	Sarafidis et al. [61], 2017, Greece	Case-control (prospective, pilot)	Urine	1H-NMR + LC-MS/MS (Targeted & Untargeted)	16 LOS (9 confirmed + 7 possible)	16	Model validity: Robust Q² (≈0.75–0.77); per-metabolite ROC in supplement (not all extractable here).	Confirmed by blood culture OR ≥ 2 clinical + ≥2 lab criteria (EMA 2010)
S7	Mardegan et al. [62], 2021, Italy	Case-control (prospective)	Urine (untargeted) & Plasma (targeted)	UPLC-QToF-MS (untargeted), UPLC-TQ-MS (targeted)	15 (EOS, preterm)	15 matched preterms		EOS within 72 h; ≥ 2 clinical + ≥2 lab signs (EMA 2010); 1 positive blood culture
S8	Georgiopoulou et al. [63], 2022, Greece	Case-control (prospective, 4-arm design)	Urine	Untargeted 1H-NMR	34 total (23 EOS, 11 LOS), preterm	37 (14 NICU preterm, 23 healthy preterm)		EOS: < 72 h; LOS: > 72 h; confirmed by blood culture or CRP with symptoms
S9	Wang et al. [10], 2023, China	Case-control	Serum	LC-MS (Untargeted)	30, term	30	AUC= 0.860 (95 %CI 0.765–0.955); prolyl-hydroxyproline alone AUC 0.83; correlations with CRP/PCT noted.	Confirmed by CRP, PCT, blood culture, x-ray, etc.
S10	Bian et al. [9], 2024, China	Case-control	Serum	LC-MS (Untargeted)	43 (25 INF + 18 SI), term	25	AUC= 1.00 (in-sample); per-metabolite AUCs 0.74–0.97; caution re overfitting.	Blood culture / CRP / PCT positive
S11	Liu et al. [17], 2024, China	Case-control (prospective, matched)	Stool	Untargeted LC-MS	18 (preterm, LOS only)	18 (matched preterm controls)	R² = 0.81, Q² < 0 AUC > 0.7 for several metabolites	LOS > 3 days; confirmed by culture or ≥ 2 lab criteria + symptoms

1H-NMR - Proton nuclear magnetic resonance, GC-MS - Gas Chromatography-Mass Spectrometry, UPLC-MS/MS - Ultra-Performance Liquid Chromatography-Tandem Mass Spectrometry, LC-MS/MS - liquid chromatography-tandem mass spectrometry, LC-MS - Liquid Chromatography-Mass Spectrometry, UPLC-QToF-MS - Ultra-Performance Liquid Chromatography coupled with Quadrupole Time-of-Flight Mass Spectrometry, UPLC-TQ-MS - Ultra-Performance Liquid Chromatography-Triple Quadrupole Mass Spectrometry, EOS – early onset sepsis, LOS – late onset sepsis, INF - sepsis group with sepsis without complications, SI - sepsis along with comorbidities group, PICU – pediatric intensive care unit, NICU – neonatal intensive care unit, PRISM III-APS - Pediatric Risk of Mortality III-Acute Physiology Score, CRP – C reactive protein, PCT – procalcitonin.

Across the 11 studies, the designs varied notably by prospective versus retrospective, case-control versus cohort, focus on early onset sepsis (EOS) versus late onset sepsis (LOS), composition of term versus preterm, and biofluid (serum, urine, dried blood spot, and stool). To facilitate cross-study interpretation, we provide a structured overview (Table 1), which stratifies by design, onset category (EOS/LOS), maturity (term/preterm), and biofluid. We also indicate whether targeted validation was performed and if external validation cohorts were used.

**Fig. 2:**
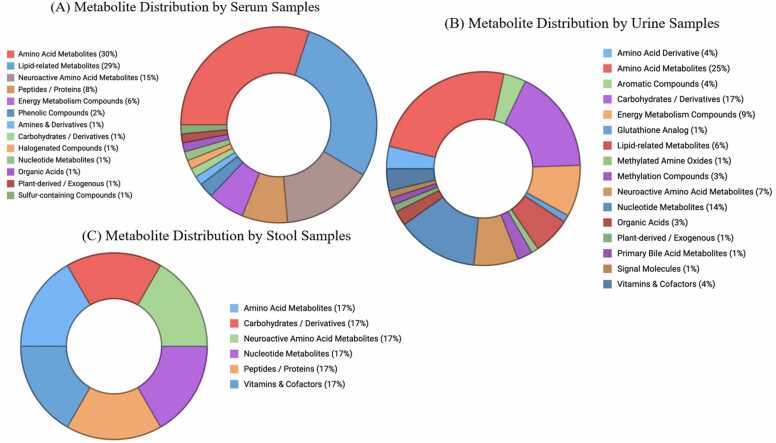
Distribution of the most frequently reported metabolite across biofluids. Note: Metabolite distribution across different biofluids in neonatal sepsis studies: serum (A), urine (B), and stool (C). Serum and urine are the most commonly analyzed matrices, while stool-based analyses are less frequent but represent an emerging area of interest.

Overall, an initial list of 153 metabolites was identified across all biofluids. After duplicate removal, 129 unique metabolites remained. Of these, eight metabolites were identified as frequently reported across studies: glucose, lactate, taurine, creatinine, glycine, acetate, tyrosine, and valine. Glucose and lactate were consistently reported as elevated in neonatal sepsis, suggesting systemic metabolic stress characterized by altered energy metabolism. Taurine and glycine exhibited mixed directional changes, indicating context-dependent metabolic dysregulation, which may be influenced by factors such as timing, severity, and differences in the source biofluid. Fig. 2 illustrates the distribution of frequently reported metabolites across different biofluids (serum, urine, and stool) in neonatal sepsis studies. Serum and urine are the most commonly used matrices for metabolomic profiling. Serum-based analyses predominantly identify amino acids, energy metabolites, and lipid derivatives, while urine-based analyses identify filtered and excreted metabolites. Urine provided complementary data on filtered and excreted metabolites. Although underrepresented, stool-based analyses highlighted the emerging interest in gut microbiota-derived metabolites. Among all the metabolites, as shown in Table 2, eight metabolites frequently reoccurred with either consistent directional or variable changes. Serum and urine dominated the analysis of biomarkers, while stool metabolites were underrepresented. This highlights a potential research gap, especially given the emerging understanding of the interactions between the gut microbiome and metabolome in neonatal sepsis [17].

**Table 2:** Most frequently observed metabolites across studies.

Metabolite	Study Count	Directionality	Biofluids
Glucose	4	↑	Serum, Urine
Lactate	3	↑	Serum, Urine
Taurine	3	↑, ↓	Urine
Creatinine	2	↑, ↓	Serum, Urine
Glycine	2	↑, ↓	Serum, Stool
Acetate	2	↑, ↓	Serum, Urine
Tyrosine	2	↑	Serum
Valine	2	↑	Serum, Urine
Phenylalanine	2	↑	Serum, Urine

When available, we extracted fold changes, odds ratios, and area under the curve (AUC) values for candidate metabolites. These metrics and their 95 % confidence intervals (CIs) are presented in Table 1 to complement the frequency counts.

After classifying the metabolites into 21 groups, ORA was performed. The results of the ORA and Fisher’s test are presented in Table 3.

**Table 3:** The results of ORA and Fisher’s test.

**Grouped Class**	**Enrichment Ratio**	***P*****-value**	**Interpretation**
Organic Acids	1.6	0.252	Suggestive enrichment; needs validation
Vitamins & Cofactors	1.6	0.158	Suggestive enrichment; needs validation
Amino Acid Derivative	1.6	0.252	Suggestive enrichment; needs validation
Sulfur-containing Compounds	1.58	0.634	Low representation; limited statistical or biological relevance
Methylated Amine Oxides	1.58	0.634	Low representation; limited statistical or biological relevance
Amines & Derivatives	1.58	0.634	Low representation; limited statistical or biological relevance
Phenolic Compounds	1.58	0.634	Low representation; limited statistical or biological relevance
Halogenated Compounds	1.58	0.634	Low representation; limited statistical or biological relevance
Carbohydrates / Derivatives	1.21	0.232	Suggestive enrichment; needs validation
Lipid-related Metabolites	1.14	0.280	Suggestive enrichment; needs validation
Amino Acid Metabolites	1.05	0.427	Frequent biomarkers but diluted by many non-biomarkers; functionally important but not statistically enriched
Neuroactive Amino Acid Metabolites	1.02	0.566	Frequent biomarkers but diluted by many non-biomarkers; functionally important but not statistically enriched
Energy Metabolism Compounds	0.85	0.831	Small number of biomarkers; may be relevant but underpowered for enrichment
Plant-derived / Exogenous	0.79	0.868	Low representation; limited statistical or biological relevance
Methylation Compounds	0.79	0.868	Low representation; limited statistical or biological relevance
Nucleotide Metabolites	0.59	0.987	Small number of biomarkers; may be relevant but underpowered for enrichment
Peptides / Proteins	0.52	0.975	Low representation; limited statistical or biological relevance
Signal Molecules	0	1	Low representation; limited statistical or biological relevance
Primary Bile Acid Metabolites	0	1	Low representation; limited statistical or biological relevance
Aromatic Compounds	0	1	Low representation; limited statistical or biological relevance
Glutathione Analog	0	1	Low representation; limited statistical or biological relevance

The analysis has shown high ER for the following classes: organic acids, vitamins and cofactors, and amino acid derivatives (all three groups having an ER of 1.6), with a *p*-value above 0.15. While those classes do exhibit high representation, each class had only a couple of reported metabolites, significantly reducing the statistical power of the class analysis.

Although no class was identified to be overrepresented, several metabolite classes were shown to carry substantial interest as potential biomarkers. Amino acid metabolites were among the most frequently investigated and reported as biomarkers (n = 24). The enrichment ratio was moderate (ER = 1.05) and did not reach statistical significance (*p* = 0.427). Having the highest biomarker rate among all other classes, the low enrichment of this class was probably diluted due to the high number of non-biomarkers. However, some amino acids, including taurine, glycine, and phenylalanine, were among the most consistently identified. Taurine and glycine have exhibited mixed regulation, with two studies indicating downregulation and one study showing upregulation for taurine and the opposite trends for glycine. Phenylalanine, on the other hand, has mostly been shown to be upregulated (two studies for upregulation and one for downregulation). The observed results suggest prominent changes in amino acid metabolism in sepsis-related metabolic dysregulations, although these changes do not reach statistical significance.

The class of lipid-related metabolites, including acylcarnitines, lysophospholipids, and glycerophospholipids, has also shown modest enrichment (ER = 1.14, *p* = 0.280), warranting further investigation for potential use as biomarkers. Containing fewer biomarkers (n = 14) per class, the analysis of lipid-related metabolites revealed a higher ER due to the smaller number of entries assigned as non-biomarkers compared to other groups.

The ORA for energy metabolism compounds yielded an enrichment ratio of 0.85 with a *p*-value of 0.831. The class contained highly sepsis-associated markers such as lactate and creatinine. While our results did not reach conventional significance, given the strong evidence for the diagnostic and prognostic abilities of these markers, their varying distribution as both biomarkers and non-biomarkers across the studies likely made it difficult to reflect their involvement statistically. The same observation has been made for the class of carbohydrates and derivatives (ER = 1.21, *p* = 0.232). Among all other metabolites, glucose was reported to be upregulated in 5 studies, making it the most consistent metabolite.

Some metabolites (e.g., carnosol, dihydrovaltrate) were categorized under the “plant-derived/exogenous” class because they were not part of any of the proposed metabolic pathways and were linked to external exposure (maternal diet, environment); thus, they should be investigated cautiously.

Due to the sparse and heterogeneous nature of the datasets, the ORA results are presented alongside enrichment ratios and exact p-values but are explicitly down-weighted in the conclusions. We performed a leave-one-study sensitivity check for recurrent metabolites (≥ three cohorts) and flagged findings that were robust versus study-specific.

## 6 Risk of bias

We independently assessed risk of bias with consensus resolution using an adapted QUADAS-2 for diagnostic discovery/validation and a ROBINS-I for capturing observational biases. Full domain judgments and notes appear in Supplementary Tables S1 and S2.

Across the eleven studies, QUADAS-2 (See Supplementary Table S1) showed that patient selection was often at high risk due to small, single-center case-control sampling and potential spectrum effects. Index test bias was low-moderate overall, but it was weakened by infrequent reporting of analytic blinding and leakage-safe cross-validation. Reference standards were at moderate risk due to mixed culture-proven and clinical sepsis definitions. Flow and timing were generally at low risk, with sampling close to decision points. Applicability concerns were moderate due to preterm-heavy NICU populations, platform heterogeneity, and center-specific sepsis criteria.

For ROBINS-I (Details are in Supplementary Table S2), the overall risk was moderate for most studies, with serious risk in two studies: the newborn screening registry (residual confounding and outcome misclassification) and a small machine learning (ML)-based serum panel (overfitting and selected reporting). Typical patterns included moderate confounding and selection bias, low deviations and missingness, and low-to-moderate outcome measurement bias, as well as moderate-to-serious selection bias in the reported results, where multiple pipelines were explored without external validation.

## 7 Limitations of a formal meta-analysis

We did not perform a quantitative meta-analysis because the body of literature is highly heterogeneous in terms of case definitions (culture-proven versus clinically adjudicated; end-of-study versus loss-of-study), sampling time points (birth/initial evaluation, day three, convalescence), matrices (urine, serum/plasma, dried blood spot), and analytical platforms (NMR versus varied LC-MS workflows). Additionally, most studies report AUCs or Q² without threshold-based 2 × 2 data, which precludes pooled sensitivity/specificity or summary ROC modeling. Designs are typically small, single-center case-control studies with internal validation, which is often optimistic, and risk of spectrum bias; external validation and calibration are rare. Preanalytical and clinical factors (e.g., gestational age, feeding/total parenteral nutrition, antibiotics, and comorbidities), batch effects, and divergent data processing (e.g., normalization, missing data, and multiple testing) further limit comparability. Feature identification is inconsistent, which complicates metabolite-level harmonization and pathway alignment. Longitudinal dynamics (D0 - D3 - D10) introduce within-subject correlation and time-varying effects that aren't consistently modeled. Selective reporting and small-study effects remain potential issues, which could lead to misleading pooled estimates. Additionally, the combination of small case-control designs, heterogeneous pre-analytics, and NICU-specific exposures (parenteral nutrition, antibiotics, and carnitine/lipid supplementation) increases the risk of false positives (e.g., treatment-driven metabolite shifts) and false negatives (e.g., signal dilution after overcorrection or misclassification), particularly in untargeted workflows with incomplete metabolite identification.

## 8 Discussion

The objective of this systematic review was to evaluate the current state of metabolomics research in neonatal sepsis and to identify potential metabolic biomarkers that could enhance diagnostic accuracy in this population. By synthesizing data from 11 studies using various analytical platforms and biofluids, we identified recurring metabolites and metabolite classes associated with neonatal sepsis. Despite differences in methodology and study design, several consistent findings emerged, offering valuable insights into the pathophysiology of neonatal sepsis and the feasibility of metabolomics-based diagnostics.

Sepsis induces a hypermetabolic state, triggering catabolic pathways to meet increased energy demands [18]. The consistent upregulation of metabolites central to protein catabolism reflects the enhanced requirement for cellular survival through proteolysis, generating gluconeogenic substrates [19]. Several studies have found that glucose and lactate levels increase in response to stress-induced glycolytic reprogramming and anaerobic metabolism [20]. Hyperglycemia in neonatal sepsis likely results from increased hepatic gluconeogenesis and insulin resistance, both of which are part of the adaptive metabolic response to inflammation and infection [18], [21]. Elevated lactate levels are a hallmark of hypoperfusion and mitochondrial dysfunction. They reflect impaired oxygen utilization at the tissue level, a finding consistent with the pathophysiology of sepsis in both adult and neonatal populations [22].

Elevated creatinine and lactate levels also may indicate disruption of central energy-generating pathways, including glycolysis, the TCA cycle, and oxidative phosphorylation [23]. Elevated creatinine levels may indicate increased muscular catabolism and early renal dysfunction, which are common consequences of systemic inflammation and hypotension in sepsis [24]. These results align with the metabolic reprogramming previously reported in septic patients, in which aerobic pathways are downregulated in favor of anaerobic metabolism to support the heightened energy demands of immune cell activation [14], [18].

Furthermore, changes in carbohydrate derivatives (e.g., glucose and acetate) reflect hepatic metabolic adaptations to infection. Acetate, which can be derived from pyruvate, fatty acids, or amino acids, serves as a short-chain fatty acid and signaling molecule. Altered acetate levels in neonatal sepsis may indicate disruptions in host metabolism and microbiome-derived metabolite profiles [17].

The existing literature supports the role of amino acid metabolism pathways in both innate and adaptive immunity [25]. The consistent detection of phenylalanine, tyrosine, valine, and taurine, along with variable levels of glycine, underscores the complex disruption of amino acid homeostasis. In particular, elevated phenylalanine has been associated with hepatic dysfunction and impaired phenylalanine hydroxylase activity in sepsis [26]. The interplay between aromatic amino acids and immune function is well documented. Phenylalanine and tyrosine are precursors of catecholamines that play essential roles in immune modulation and neurotransmitter synthesis [25], [27]. Abnormalities in taurine and glycine metabolism suggest disruptions in antioxidant defense and cytoprotection, as both compounds play crucial roles in maintaining cellular redox balance and osmoregulation [28].

Notably, the directionality of some amino acids, especially taurine and glycine, was inconsistent across studies. This variation may be attributed to differences in sample timing (e.g., early versus late sepsis), disease severity, treatment interventions (e.g., parenteral nutrition), and the biofluid compartment analyzed. For instance, glycine levels may decrease due to increased consumption during oxidative stress or increase in response to catabolic processes and impaired renal clearance [29]. Many neurotransmitter pathways closely intersect with amino acid metabolism, potentially reflecting microglia-induced neuroinflammation or blood-brain barrier dysfunction that may lead to neurodevelopmental sequelae [30]. Thus, these findings underscore the importance of developing neuroprotective strategies for neonates with sepsis.

Alterations in lipid-related metabolites, including acylcarnitines and lysophospholipids, suggest dysregulated β-oxidation and mitochondrial dysfunction [29]. In particular, the accumulation of acylcarnitines has been observed in multiple sepsis models and reflects the impaired transport of fatty acids into mitochondria, a process that is tightly regulated by carnitine-dependent pathways [11]. These changes are exacerbated by inflammatory signaling, which directly impairs mitochondrial enzymes and electron transport chain activity. The resulting energetic failure contributes to multiorgan dysfunction, a common terminal pathway in severe sepsis [31].

Lysophospholipids, on the other hand, are bioactive lipids involved in cell signaling, immune modulation, and membrane remodeling. Their dysregulation in sepsis may reflect increased phospholipase activity due to systemic inflammation or cell damage [29]. Due to the limited number of studies focusing on these lipid classes, future research utilizing targeted lipidomics is necessary to validate their diagnostic and prognostic value.

The reviewed studies analyzed multiple biofluids, including serum, urine, and stool. Each biofluid reflects a different aspect of neonatal metabolism. Serum provides an overview of circulating metabolites and immediate metabolic responses to infection, while urine reflects renal filtration and the excretion of metabolic byproducts over time [9]. Though underrepresented in the literature, stool metabolites provide insight into host-microbiota interactions.

Notably, one study [17] included stool-based metabolomics. This study provides an innovative approach to investigating the role of the gut microbiome in neonatal sepsis pathogenesis. Gut-derived metabolites, such as short-chain fatty acids, bile acid derivatives, and tryptophan metabolites, are recognized as modulators of systemic immunity [30]. Given the high prevalence of gut dysbiosis in preterm neonates, integrating microbiome and metabolome data could lead to the discovery of novel, noninvasive biomarkers and therapeutic targets.

Urine and stool offer noninvasive collection methods, which are especially attractive in fragile neonatal populations. However, metabolite concentrations in these fluids can be influenced by hydration status, renal function, and intestinal transit time. Therefore, careful standardization in study protocols is necessary.

It is important to mention that no single metabolite can be considered highly sensitive or specific for sepsis. For this reason, it would be more effective to organize a metabolic panel of various disrupted pathways to accurately depict the host response to sepsis.

Mapping recurrent metabolites to KEGG/Reactome highlights convergent perturbations in glycolysis-TCA-OXPHOS, branched-chain amino acid catabolism, and lysophospholipid remodeling [32]. While our ORA was underpowered, the directionally consistent increases in glucose and lactate, elevations in phenylalanine/tyrosine, and signals in acylcarnitines align with canonical sepsis bioenergetic and mitochondrial stress pathways [33], [34]. We therefore interpret pathway hits as hypothesis-generating and propose targeted panels spanning these axes for future validation.

ORA failed to identify any statistically significant enrichment of metabolite classes, likely due to the limited number of studies and the heterogeneity of metabolite identification methods. Nevertheless, several classes, including organic acids, amino acid derivatives, and vitamins/cofactors, showed enrichment ratios exceeding 1.5, suggesting potential biological relevance.

The lack of statistical significance should not negate the clinical importance of these findings. For instance, although amino acid metabolites were not statistically significantly overrepresented, they were among the most frequently reported classes. This finding is consistent with the knowledge that sepsis causes extensive protein catabolism and amino acid mobilization to support gluconeogenesis, immune cell proliferation, and antioxidant responses [25], [27].

The statistical challenges encountered in the ORA further emphasize the need for larger, well-powered studies with harmonized methodologies. Integrating metabolomics datasets across studies, employing standardized biofluid collection protocols, and adopting consistent definitions of neonatal sepsis are essential steps for deriving robust conclusions.

## 9 Critical appraisal of metabolite signals

Across studies, only a few signals appear robust, while others are plausibly confounded by routine NICU exposures. Signals that recur in at least three independent cohorts include glucose and lactate, both of which are directionally increased and biologically consistent with stress hyperglycemia, glycolytic reprogramming, tissue hypoperfusion, and mitochondrial dysfunction. Taurine (three studies) recurs with mixed directionality, likely reflecting timing, matrix, renal handling, and intervention effects. Several candidates, however, are susceptible to confounding factors. For example, total parenteral nutrition can elevate circulating glucose and selected amino acids, lipid emulsions and carnitine supplementation can influence acylcarnitines, and broad-spectrum antibiotics can alter gut-derived metabolites, such as short-chain fatty acids and bile acid derivatives, especially in stool-based profiles [35], [36]. Comorbidities, such as renal dysfunction, can also alter creatinine and amino acid clearance [37]. In terms of biological plausibility, lipid classes, particularly acylcarnitines and lysophospholipids, map closely to known sepsis pathophysiology (e.g., β-oxidation impairment, membrane remodeling, and inflammatory signaling) [38]. However, several plant-derived or exogenous features likely reflect maternal diet or environment and should be interpreted cautiously. Overall, the most credible neonatal sepsis markers in our analysis are glucose and lactate, which are directionally consistent and mechanistically anchored. Next are acylcarnitines and lysophospholipids, which are pathway-level indicators of mitochondrial stress. Taurine and glycine are still tentative, pending studies that explicitly control for nutrition, antibiotics, renal function, and sampling time.

## 10 Clinical implications

Metabolomics has the potential to revolutionize the diagnosis of neonatal sepsis by providing a comprehensive biochemical snapshot of the host’s real-time physiological state [39]. However, despite the promising metabolic signatures identified in various studies, translating these findings into clinical practice remains challenging [40]. One of the most significant barriers is the considerable heterogeneity in study designs. Across the reviewed literature, there was significant variation in gestational age at inclusion, sepsis definitions, timing of sample collection relative to disease onset, and metabolomics platforms. These inconsistencies hinder direct comparisons and synthesis of the findings into a unified diagnostic framework.

To be useful at the bedside, metabolomics outputs should be presented as structured observations in the electronic health records (EHR) and incorporated into rule- or model-based Clinical Decision Support Systems (CDSS) [41], [42]. One pragmatic approach is to publish each validated metabolite or composite risk score with clear units, reference ranges, sample type/time, and provenance. Mapping to shared terminologies enables computable triggers (e.g., automatic sepsis risk alerts when thresholds are crossed) and ensures that alerts can be combined with vitals, labs, and antimicrobial orders. Integrating the outputs into clinician workflows (e.g., flowsheets, order sets, and sepsis pathways) with human factors safeguards (e.g., clear thresholds, rationale, and explainability of "why this fired") reduces alert fatigue and supports actionability.

Another critical limitation is the scarcity of external validation [13]. While several studies have proposed potential biomarkers, few have confirmed their performance in independent neonatal cohorts. This lack of reproducibility is a key obstacle to clinical implementation, especially in neonatology, where high diagnostic accuracy is essential. Compounding this issue is the absence of established reference ranges and diagnostic thresholds for most identified metabolites, which makes it challenging to interpret metabolomic profiles within a clinically actionable context [43]. Unlike well-characterized inflammatory markers such as CRP or PCT, the metabolites identified through untargeted analyses often lack interpretative cut-offs. To date, no metabolomics-based panel has achieved regulatory approval for the diagnosis of neonatal sepsis, highlighting the need for robust, multicenter validation.

To ensure that discovery data remains FAIR (findable, accessible, interoperable, and reusable) across institutions, it should use open, vendor-neutral formats (e.g., mzML/nmrML for raw spectra), capture study context, and apply any universal standards for sample preparation, acquisition, and identification [44], [45]. For clinical deployment, derived features and model inputs should be harmonized with an enterprise data model. The minimum reporting requirements should include analytical parameters (e.g., instrument, quality control [QC] strategy, limit of detection [LoD]/limit of quantification [LoQ], and drift correction), pre-analytical parameters (e.g., matrix and timing versus antibiotics/total parenteral nutrition [TPN]), and governance parameters (e.g., consent scope, data retention, and audit trails). This standardization enables reproducibility, multi-site validation, and safe reuse for post-market surveillance [46].

Furthermore, metabolite concentrations are influenced by various external and biological factors, including nutritional intake, parenteral nutrition, medication exposure, renal and hepatic function, and comorbidities such as necrotizing enterocolitis or intraventricular hemorrhage [47]. These variables introduce noise into metabolomic data, underscoring the need for careful control of confounding variables in research and clinical settings.

Due to these complexities, it is unlikely that a single metabolite can serve as a definitive biomarker for neonatal sepsis. A more realistic and promising approach is to develop composite biomarker panels that reflect multiple disrupted metabolic pathways simultaneously. Such panels could offer enhanced sensitivity and specificity compared to individual markers [48]. Recent advances in machine learning and artificial intelligence may further improve the diagnostic power of metabolomic data by identifying metabolite patterns or clusters that best distinguish septic from non-septic neonates [49]. Concurrently, integrating metabolomic findings with other clinical and laboratory parameters could usher in a new era of precision diagnostics. In this era, metabolic fingerprints would be used for early detection, disease stratification, risk assessment, and therapeutic monitoring.

For metabolomics to become part of routine neonatal care, however, further progress is needed in assay standardization, cost-effectiveness, turnaround time, and the development of user-friendly interpretation tools that can be readily adopted by neonatologists and clinical laboratories.

## 11 Future directions

The field of metabolomics in neonatal sepsis is at a promising yet pivotal juncture. While substantial progress has been made in identifying candidate biomarkers, future efforts must address current limitations and harness the full potential of this powerful analytical approach.

A critical first step is to standardize methodologies across studies. Harmonizing sepsis definitions, biofluid selection, sampling protocols, and analytical workflows is essential to ensuring the comparability of results and enabling meta-analytic approaches. Uniform criteria for identifying and reporting potential biomarkers will enable more robust cross-study validation and facilitate the development of shared databases, thereby accelerating the discovery and translation of findings [50].

Beyond methodological consistency, the next phase of research should prioritize large-scale, multicenter, prospective studies that encompass diverse neonatal populations, including preterm and term infants [12]. These studies must also reflect the wide variability in clinical presentations and resource settings. These studies are crucial for external validation of proposed biomarkers and for understanding the influence of demographic, genetic, and environmental factors on the neonatal metabolome. Developing metabolite panels that are robust across populations and geographies would be a significant step toward achieving clinical applicability. Furthermore, a systems biology approach is likely to benefit the future of sepsis diagnostics. To build a more comprehensive molecular profile of sepsis, metabolomics should be integrated with other omics modalities, such as genomics, transcriptomics, proteomics, and microbiomics [51], [52]. This integration will provide a multidimensional understanding of host-pathogen interactions and may reveal novel biomarkers or therapeutic targets that would be missed by analyzing each dataset in isolation. This approach is especially important for neonates, whose immune responses and metabolic processes are still developing and may differ substantially from those of older children or adults.

Another promising area of future research is the role of the gut microbiota in shaping the neonatal metabolome [53]. Though currently underexplored, stool-based metabolomics may offer unique insights into the interplay between microbial dysbiosis and systemic inflammation in sepsis. Given the emerging evidence that certain gut-derived metabolites can modulate immune responses and systemic homeostasis, expanding this area of research may lead to new possibilities for diagnosis and intervention, potentially through microbiome-targeted therapies [17].

Beyond discovery, stool metabolomics requires standardized collection windows (e.g., ±12–24 h around the onset of sepsis), dietary and parenteral nutrition logs, and the co-profiling of the microbiome, bile acid, and tryptophan pathways to mechanistically link gut-derived metabolites to the immune response and barrier function in neonates [54].

Technological advancements will also be crucial in translating metabolomic insights to the bedside. Portable, high-throughput mass spectrometry systems and microfluidic-based biosensors are being developed and may one day enable point-of-care metabolic profiling in neonatal intensive care units [55]. These tools could enable the real-time assessment of disease progression, therapeutic efficacy, and risk of complications, which would dramatically transform the timeliness and personalization of care in sepsis management.

As the field moves closer to clinical translation, it will be crucial to address ethical and regulatory considerations proactively [56]. Informed consent processes must be adapted to accommodate the complex nature of omics research, especially when working with vulnerable populations, such as neonates. Additionally, policy frameworks must incorporate considerations around data security, privacy, and equitable access to metabolomic technologies to ensure responsible and inclusive implementation.

Given the vulnerability of NICU populations, governance should include data minimization, secure linkage, and equitable access safeguards. Multi-site data sharing can use de-identified, consent-appropriate repositories with auditable access controls, and clear policies on secondary use.

In summary, the future of metabolomics in neonatal sepsis depends on refining biomarker discovery and building the technological, clinical, and ethical infrastructure necessary for precision diagnostics for all neonates. Through collaborative efforts across disciplines, metabolomics has the potential to transform our approach to the early diagnosis and management of one of the most challenging conditions in neonatology.

## 12 Strengths and limitations

Our study provides a systematic overview of the metabolites reported to be altered in neonatal sepsis across various studies. Bridging the data across isolated studies helps identify patterns that might have been left unseen in individual study reports. The analysis of directionality, as well as the class-level associations, provides an additional context for the ability of specific metabolites or metabolite families to serve as a diagnostic tool.

While the study provides an overview of metabolite changes observed in neonatal sepsis cases, it carries several limitations that should be recognized. The first and most notable limitation is the limited statistical power of both analyses used. The sample size within each metabolite class was just enough to be eligible for a modest statistical analysis, severely restricting the sensitivity of the tests. Although metabolites were grouped in an attempt to increase statistical power, this may have profoundly masked the underlying, subtle impact of a specific metabolic pathway under the broader and more general category. Additionally, compared to other metabolites, some may not have a strong association with the class to which they have been grouped, potentially skewing the enrichment analysis. The studies included in this review demonstrate significant heterogeneity in study populations (preterm vs. full-term neonates, early vs. late-onset sepsis), sepsis definitions, and detection techniques. Although our study failed to derive statistical significance for the metabolites highly associated with sepsis (e.g., lactate), we acknowledge their strong clinical relevance and highlight the need for an analysis that captures both class-level and metabolite-specific compound analysis. This approach would improve the validation of specific metabolites and better assess their diagnostic and prognostic value.

## 13 Conclusion

This systematic review provides a comprehensive synthesis of the current evidence on metabolomic alterations in neonatal sepsis. It highlights the potential of this emerging field, as well as its limitations. Several metabolite signatures were consistently associated with sepsis-related pathophysiology across a diverse range of studies, most notably those involving amino acid metabolism, energy substrates, and lipid derivatives. Glucose and lactate were consistently elevated, reflecting metabolic stress and anaerobic adaptation. Meanwhile, aromatic and sulfur-containing amino acids exhibited complex regulatory patterns, suggesting their role in immune modulation and oxidative balance.

While no single metabolite class achieved statistically significant enrichment across all studies, the frequent recurrence of specific metabolite families highlights their potential diagnostic relevance. However, the variability in study methodologies, sepsis definitions, and biofluid types remains a substantial barrier to standardization and clinical translation.

The findings suggest that neonatal sepsis is not characterized by a single biochemical disturbance, but rather by a constellation of metabolic shifts involving multiple interconnected pathways. This complexity argues against relying on a single biomarker and instead advocates for developing multiplex panels that capture the multidimensional nature of the host response. Integrating these metabolomic profiles with machine learning and multi-omics approaches could improve diagnostic accuracy, enable earlier identification, and inform personalized treatment strategies.

Metabolomics is a highly informative, yet still maturing, tool in the diagnostic arsenal for neonatal sepsis. Future research should focus on large-scale, prospective validation studies, harmonization of methodologies, and integration with systems biology frameworks. Only through these coordinated efforts can metabolomics evolve from a promising investigational strategy into a routine clinical modality that can transform neonatal sepsis diagnostics and improve outcomes for this vulnerable population.

## Data sharing statement

This review analyzed published data. Extraction forms, effect-size calculations, HMDB mappings, and ORA scripts (with fixed seeds and environment files) will be deposited in a public repository upon acceptance, enabling full computational reproducibility.

## Author statement

Each named author has substantially contributed to conducting the underlying research and drafting this manuscript. Additionally, the named authors have declared no conflict of interest, financial or otherwise. All authors approved the submission to CSBJ. The manuscript has not been submitted to another journal prior to this submission.

## CRediT authorship contribution statement

**Olga Mironova:** Writing – review & editing, Investigation, Formal analysis. **Abduzhappar Gaipov:** Writing – review & editing, Formal analysis. **Vitaliy Sazonov:** Writing – review & editing, Writing – original draft, Validation, Supervision, Project administration, Methodology, Funding acquisition, Formal analysis, Data curation, Conceptualization. **Zhalaliddin Makhammajanov:** Writing – review & editing, Writing – original draft, Visualization, Methodology, Investigation, Formal analysis, Data curation. **Dinara Galiyeva:** Writing – review & editing, Resources, Formal analysis. **Aliya Baizhanova:** Writing – review & editing, Writing – original draft, Visualization, Methodology, Investigation, Formal analysis, Data curation, Conceptualization.

## Funding

This research was funded by 10.13039/501100012632Nazarbayev University under FDCRGP # 040225FD4716.

## Declaration of Competing Interest

The authors have declared no conflict of interest
