# Supplementary material for: Metabolomics in neonatal sepsis: A critical appraisal of current evidence
Source: Comput Struct Biotechnol J. 2025 Oct 18;27:4459–68. doi: 10.1016/j.csbj.2025.10.034 (PMC12590226; doi:10.1016/j.csbj.2025.10.034)
Supplement: Supplementary file 1 — Supplementary material [file mmc1.docx]

Supplementary materials

Search strategy

**Core Boolean strategy (example: PubMed/MEDLINE):**
("neonate"[MeSH Terms] OR neonat* OR newborn* OR infant*) AND (sepsis OR septicemia OR "blood stream infection" OR "late-onset sepsis" OR "early-onset sepsis" OR EOS OR LOS) AND (metabolom* OR "metabolic profiling" OR "metabolic fingerprint*" OR "metabolic signature")

**Filters**: Humans; Age: birth -1 month.

Scopus/Web of Science strings were harmonized to field tags and controlled vocabulary of each platform, and we hand-searched the reference lists of all eligible papers. The date of last search was June 25, 2025. Two reviewers independently screened titles/abstracts and full texts; disagreements were resolved by consensus or a third reviewer.

Supplementary Table S1. Detailed study-level ratings and justifications QUADAS-2

| Study ID | Citation/Short Title | RoB: Patient selection | RoB: Index test | RoB: Reference standard | RoB: Flow & timing | RoB: Overall | Applicability: Patient selection | Applicability: Index test | Applicability: Reference standard | Key support for judgment |
| --- | --- | --- | --- | --- | --- | --- | --- | --- | --- | --- |
| S1 | Mickiewicz et al. [43], (2013) pediatric serum 1H-NMR (includes neonates) | High | Moderate | Moderate | Low | Moderate | Moderate | Moderate | Moderate | Case control spectrum; PICU mix of ages; sepsis adjudication partly clinical; blinding not stated; NMR QC adequate. |
| S2 | Fanos et al. [28],(2014) neonatal urine 1H-NMR + GC-MS (EOS/LOS) | High | Moderate | Moderate | Low | High | Low | Moderate | Low | Small case control; culture status mixed; index-test blinding unclear; sampling at diagnosis appropriate; risk of overfitting limited by simple stats. |
| S3 | Bekhof et al. [44], (2015) preterm cohort (glucosuria; LONS) | Low | Low | Moderate | Low | Low | Low | Moderate | Moderate | Prospective cohort; predefined dipstick; reference partly clinical+microbiological; minimal flow issues; applicability better for screening than diagnosis. |
| S4 | Stewart et al. [45], (2016) longitudinal NEC/LOS serum proteo metabolomics | High | Moderate | Moderate | Low | High | Low | Moderate | Moderate | Very small matched case control; discovery focus; partial blinding unclear; good timing (вҲ’14/0/+14 d); potential spectrum bias. |
| S5 | Fell et al. [46],(2017) NBS registry (multi analyte predictive) | Moderate | Moderate | High | Moderate | High | High | High | High | Population registry; index=baseline NBS; reference=administrative/clinical sepsis; applicability concern for DTA; confounding substantial. |
| S6 | Sarafidis et al. [47],  (2017) neonatal LOS urine 1H-NMR + LC-MS/MS | High | Low | Moderate | Low | Moderate | Low | Low | Low | Prospective case control with longitudinal repeats; CV/permutation/QC reported; clear episode anchoring; small N. |
| S7 | Mardegan et al. [48]  (2021) preterm EOS plasma/urine UPLC-QToF-MS | High | Low | Moderate | Low | Moderate | Low | Low | Low | Birth sampling; QC and validation steps described; case control; EOS reference standard reasonable; pathway-driven validation. |
| S8 | Georgiopoulou et al. [49],(2022) preterm urine 1H-NMR (EOS/LOS) | High | Moderate | Moderate | Low | Moderate | Low | Moderate | Low | Four-group design; sampling windows specified; blinding not detailed; 1H-NMR pipeline standard; modest N per group. |
| S9 | Wang et al. [10],(2023) infant serum LC-MS + ML | High | High | Moderate | Low | High | Low | Moderate | Low | Discovery ML panel; internal CV only; potential leakage/overfit; reference standard mixed; good early sampling. |
| S10 | Bian et al. [9],(2024) infant serum LC-MS + ML | High | High | Moderate | Low | High | Low | Moderate | Low | Multi algorithm feature selection; discovery only AUC~1.00 suggests overfit risk; severity subgrouping increases complexity. |
| S11 | Liu et al. [17],(2024) preterm stool LC-MS (LOS) | Moderate | Moderate | Low | Low | Low | Moderate | Moderate | Moderate | Matched case–control; QC-based LOESS correction; VIP ≥ 1 + fold-change + p < 0.05 filters; multiple differential features and ROC models without external validation; permutation-tested OPLS-DA but negative Q² indicates potential overfit/limited out-of-sample performance |

Supplementary Table S2. Study-specific ROBINS-I decisions and rationales

| Study ID | Citation/Short Title | Confounding | Selection of participants | Classification of interventions/ exposure | Deviations from intended interventions | Missing data | Measurement of outcomes | Selection of reported result | Overall risk of bias | Key support for judgment |
| --- | --- | --- | --- | --- | --- | --- | --- | --- | --- | --- |
| S1 | Mickiewicz et al. [43], (2013) pediatric serum 1H-NMR (includes neonates) | Moderate | Moderate | Moderate | Low | Low | Low | Moderate | Moderate | Confounding by age/severity; PICU spectrum; measurement robust; selective reporting possible. |
| S2 | Fanos et al. [28],(2014) neonatal urine 1H-NMR + GC-MS (EOS/LOS) | Moderate | Moderate | Low | Low | Low | Low | Moderate | Moderate | Small N; potential confounding (GA, feeding, antibiotics); missing data minimal; discovery focus. |
| S3 | Bekhof et al. [44], (2015) preterm cohort (glucosuria; LONS) | Moderate | Low | Low | Low | Low | Low | Moderate | Moderate | Large cohort; confounding adjusted; measurement objective; low missingness; suitable analytic model. |
| S4 | Stewart et al. [45], (2016) longitudinal NEC/LOS serum proteo metabolomics | Moderate | Moderate | Moderate | Low | Low | Low | Moderate | Moderate | Very small; time varying confounding; selective reporting risk; outcome/omics measured consistently. |
| S5 | Fell et al. [46],(2017) NBS registry (multi analyte predictive) | Serious | Moderate | Moderate | Low | Low | Moderate | Serious | Serious | Serious confounding (sociodemographics, GA/SGA); administrative outcome misclassification risk; robust missing data handling reported. |
| S6 | Sarafidis et al. [47],  (2017) neonatal LOS urine 1H-NMR + LC-MS/MS | Moderate | Moderate | Low | Low | Low | Low | Moderate | Moderate | Moderate confounding; careful episode anchoring; minimal missing; outcome adjudication reasonable. |
| S7 | Mardegan et al. [48]  (2021) preterm EOS plasma/urine UPLC-QToF-MS | Moderate | Moderate | Low | Low | Low | Low | Moderate | Moderate | Birth sampling reduces reverse causation; confounding by GA/chorio; not fully controlled; strong QC. |
| S8 | Georgiopoulou et al. [49],(2022) preterm urine 1H-NMR (EOS/LOS) | Moderate | Moderate | Low | Low | Low | Low | Moderate | Moderate | Group differences (GA/weight) drive confounding; partial adjustment; measurement OK. |
| S9 | Wang et al. [10],(2023) infant serum LC-MS + ML | Moderate | Moderate | Moderate | Low | Low | Low | Moderate | Moderate | Feature selection + small N; reporting bias; confounding partially addressed; outcome adjudication moderate. |
| S10 | Bian et al. [9],(2024) infant serum LC-MS + ML | Serious | Moderate | Moderate | Low | Low | Moderate | Serious | Serious | Serious overfit/selective reporting risk; confounding not fully addressed; clear measurement protocols. |
| S11 | Liu et al. [17],(2024) preterm stool LC-MS (LOS) | High | Moderate | Moderate | Low | Moderate | Moderate | Moderate | Moderate | Preterm (<32 w) case–control with 1:1 matched subset; stool untargeted LC–MS; OPLS-DA with 200 permutations (R² = 0.81, Q² < 0) suggests limited predictive generalization; diagnostic criteria combine suspected/clinical/culture-proven; sampling at LOS onset; ROC AUC > 0.7 for several metabolites without external validation |

Figure S1: Overlap of Reported Metabolite Classes/Pathways Across Biofluids (S1–S11).


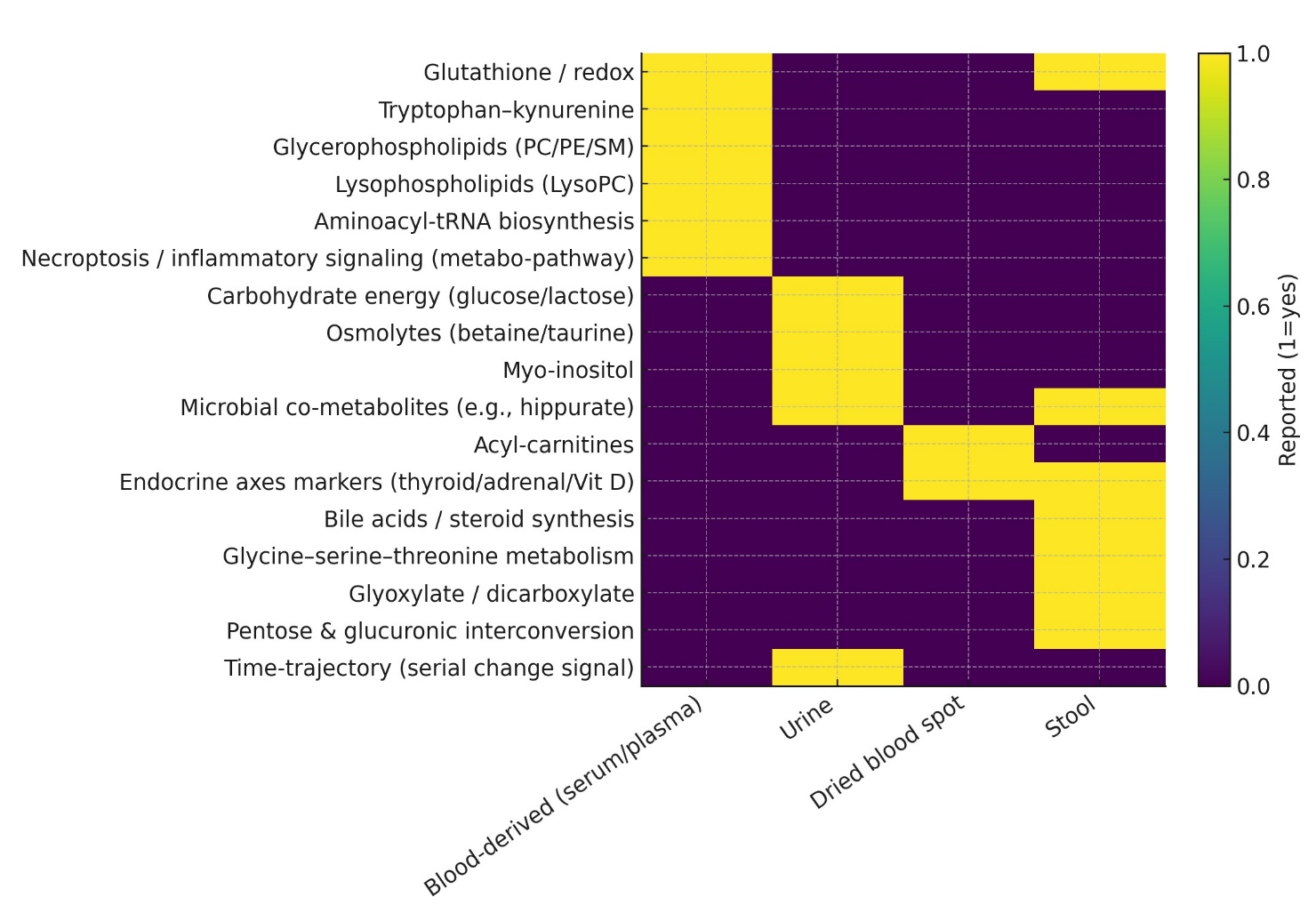


The heatmap summarizes findings from four biofluid categories: blood-derived (serum/plasma), urine, dried blood spot (DBS), and stool which are mapped to recurrent metabolite classes/pathways. Blood-derived studies (S1, S3–S5) consistently implicate glutathione/redox, tryptophan-kynurenine, and membrane lipid remodeling (glycerophospholipids and lysophosphatidylcholine [LysoPC]), with enrichment of aminoacyl-tRNA and necroptosis-linked pathways. Urine studies (S2 and S9) emphasize carbohydrate energy (glucose and lactose), osmolytes (betaine and taurine), myo-inositol, microbial co-metabolites (hippurate), and signals of serial change that track the clinical course. DBS data (S8) contribute scalable acylcarnitine and endocrine markers for early risk stratification. The stool study (S11) extends coverage to the gut compartment. It adds glycine, serine, threonine, and glyoxylate, dicarboxylate metabolism, plus bile acid and steroid pathways, as well as a vitamin D–related signal. This highlights microbe-host metabolic crosstalk in preterm LOS infants. Together, these patterns suggest the use of context-specific multi-analyte panels rather than a single universal biomarker. Examples of these panels include birth-screening flags in DBS, urine triage/monitoring, and serum/plasma confirmation.
